# Comparison between chimeric *Trypanosoma cruzi* antigens (ABBOTT PRISM Chagas assay) and local antigenic extracts in blood bank screening for Chagas disease in an endemic area for the *Triatoma dimidiata* vector

**DOI:** 10.1590/S1678-9946202567057

**Published:** 2025-08-25

**Authors:** Floribeth León Pérez, Gloria Miss-Vivas, Virginia Peña Hernández, Victor Monteon

**Affiliations:** 1 Universidad Autónoma Campeche, Facultad de Odontología, Campeche, Mexico; 2 Centro Estatal de Transfusión Sanguínea Campeche, Campeche, Mexico; 3 Universidad Autónoma Campeche, Centro de Investigaciones Biomédicas, Campeche, Mexico

**Keywords:** Trypanosoma cruzi, Blood donors, Triatoma dimidiata, Seroprevalence

## Abstract

*Trypanosoma cruzi* can be transmitted by blood transfusion in non-endemic areas, and in endemic areas the risks may be higher. We investigated the seroprevalence of anti-*T. cruzi* antibodies in 1,500 blood donors from a blood bank in an endemic area where *Triatoma dimidiata* constitutes the main vector and in 2,800 healthy young subjects. Choosing antigens is crucial in diagnostic tests as it directly influences performance across regions. We screened 1,500 blood donors in parallel for chimeric *Trypanosoma cruzi* antigens with the ABBOTT PRISM Chagas assay at the State Blood Transfusion Center and with an in-house ELISA assay using local *T. cruzi* antigenic extracts at a Biomedical Research Center laboratory. Overall, 13 *T. cruzi* isolates from the Yucatan Peninsula, Mexico, were characterized for their antigenic profiles before being used in an in-house ELISAs. All *T. cruzi* isolates shared immunodominant antigens among themselves and with South American strains (particularly those below 100 kDa). Seroprevalence of anti-*T. cruzi* antibodies in blood donors totaled 0.2% (3/1500) in the ABBOTT PRISM Chagas assay and 0.26% (4/1500) in the in-house ELISA. We found low sensitivity (33%; 95%CI 0.01710 to 0.8815), high specificity (99.8%; 95%CI 0.9941 to 0.9995), and a low kappa index (0.2). Seroprevalence in young subjects totaled 0.14 %. We found comparable seroprevalence to blood banks in large Mexican municipalities free of vector transmission. Thus, optimal public immunodominant antigens are needed to cover the broad immune spectrum induced by *T. cruzi* infection.

## INTRODUCTION

Chagas disease (CD) occurs in endemic areas and in urban centers. Its two main routes of transmissible infection occur via a causative vector or blood transfusions. Human migration may displace infected populations. *Trypanosoma cruzi* infection prevalence in immigrants depends on the region, country, and nationality^1^. Although blood screening in blood banks constitute a positive strategy to mitigate *T. cruzi* transmission and other blood borne pathogens, some endemic areas lack universal screening with highly sensitive serological method followed by confirmation from a secondary, highly specific method (according to the criteria recommended by Pan American Health Organization)^2^.

Mexico configures a CD endemic area. In the last two decades, the country has experienced waves of immigrants from other endemic Central and South American countries. Although Mexican public health policies include blood bank screening for Chagas disease via commercially available diagnostic kits, their performance, reliability, and use of these various serological platforms remains discordant and inconsistent^3^. Confirmation of chronic *T. cruzi* infection requires at least two orthogonal serological tests. However, the antigens in these assays may affect test performance and reliability^4,5^.

Commercially available ELISA diagnostic kits have undergone evaluations. They include Wiener v.3.0 and Wiener v.4.0 kits, with recombinant antigens 1, 2, 13, 30, 36 and secreted acute-phase antigen (v.3.0 uses polyclonal conjugate secondary antibodies and v.4.0, monoclonal anti-human IgG antibodies)^6,7^; the Chagatek ELISA from Lemos/Biomerieux (Santiago del Estero, Argentina), with purified *T. cruzi* antigens; the NovaLisa^®^ Chagas (*Trypanosoma cruzi*) IgG ELISA from NovaTec Inmunodiagnostica Gmbh (Dietzenbach, Germany), with *T. cruzi* recombinant antigens; the Hemagen Chagas ELISA kit (Hemagen Diagnostics, Inc., Columbia, MD), with purified antigens extracted from cultured *T. cruzi*; the Ortho *T. cruzi* ELISA test system (Ortho Clinical Diagnostics, Raritan, NJ), with the whole cell lysate antigen; the InBios Chagas Detect Plus rapid test lateral flow assay (InBios International, Inc., Seattle, WA) with TC8.2 recombinant multiepitope fusion antigen, secreted acute-phase antigen, and peptides 30 and 36 (ITC8.2 also contains two copies of the Kmp-11 peptide and peptide 1); the Abbott PRISM Chagas chemiluminescent assay (Abbott Laboratories, Abbott Park, IL), with the *T cruzi* chimeric recombinant antigens FP3, FP6, FP10, and TcF; and the Ortho ELISA Wiener Lisado, with a Tulahuen *T. cruzi* lysate. The tested assays had highest sensitivity for *T. cruzi* in blood samples from donors who were born in South America, an intermediate sensitivity for samples from Central American donors, and the lowest sensitivity from Mexican donors^3^. *T. cruzi* isolate and antigen source variability and human population heterogeneity could cause such diverse intensity and sensitivity to antigens. The trypomastigote small-surface antigen, a mucin expressed by the circulating forms of *T. cruzi* I and II, induces antibodies against it in infected subjects. A comparison of the reactivity of anti-*T-cruzi* seropositive samples from Mexico (which were tested first with antigens of Mexican strains and then with the trypomastigote small-surface antigen) showed lower concordant reactivity to samples from Argentina and Paraguay^8^. However, Mexican, Guatemalan, and CL-Brener *T. cruzi* strains provided equally good antigen sources to detect Mexican chronic Chagas cardiomyopathy (CCC) in patients. However, blood donor samples obtained a lower concordance^9,10^. Thus, Mexican patient samples require careful evaluations; particularly at blood banks because antibody titers indicating the presence of infection are generally close to the level of detection.

In Mexico, and particularly in the Yucatan Peninsula, *Triatoma dimidiata* (Latreille, 1811) configures the main vector species and *T. cruzi* I is the most prevalent^11-13^. Despite 90% of the Mayan population in rural areas knowing the vector and 65% of them having experienced bites from triatomine insects, seroprevalence in rural communities is low (4.8%), and only 6% of the rural population received a diagnosis of dilated myocardiopathy in regional hospitals. The most common diagnosis associated with dilated cardiomyopathies refer to hypertension and ischemic cases, and only 7.1% of patients were classified as having dilated CCC^14-16^.

Updated studies on seroprevalence of anti- *T. cruzi* antibodies in Mexican blood bank donors from 2014 to 2024 reported a seroprevalence from 0.12 % to 0.23% in blood banks in Mexico City and the Mexico State, respectively^17,18^. However, the most recent reported seroprevalence of anti-*T. cruzi* in blood banks in endemic areas, such as the Yucatan Peninsula, totaled 0.7% in 2011 at the Central Blood Bank of Instituto Mexicano del Seguro Social^19^.

This research seeks to update the seroprevalence of *T. cruzi* in a blood bank from an endemic region where *T. dimidiata* constitutes the main vector. We evaluated and compared two serological testing methods: commercial assays employed at the State Blood Transfusion Center (which do not utilize Mexican *T. cruzi* antigens) and an in-house ELISA with autochthonous *T. cruzi* isolates. We also compared the observed seroprevalence in this blood bank with that in young subjects.

### Ethics

This project was approved by Comite de Investigacion de Direccion General de Posgrado e Investigacion at Universidad Autonoma Campeche (opinion nº 17/UACAM/2024). All blood donors and students were informed of this study, agreed to participate in it, and signed a written informed consent. Identification remained confidential.

## MATERIALS AND METHODS

### Workflow

Initially, 13 *T. cruzi* isolates from the Yucatan Peninsula Mexico were evaluated for their antigenic profiles via Western blot analysis. Sera samples from patients with CCC and from blood donors (who had been tested positive by two techniques) were used for this study. This crucial step preceded the selection of the optimal *T. cruzi* isolates for an in-house ELISA at the Biomedical Research Center laboratory.

In this study, 1,500 blood donors were screened, independently, in two laboratories. At the State Blood Transfusion Center (CETS), the blood donors are screened for Chagas disease using chimeric *Trypanosoma cruzi* antigens (ABBOTT PRISM Chagas assay). This assay uses FP3, FP6, FP10, and TcF chimeric recombinant antigens. On the same day, a 200-µL aliquot of each serum sample from all blood donors was sent to CIB, in which an in-house ELISA with a crude antigen extract from a regional *T. cruzi* isolate was used to screen donor samples.

All initially reactive serum samples were retested at CETS. A sample was considered reactive and cleared for discharge if both analyses yielded positive results above cut-off.

Confirmation takes place outside the Blood Bank. Instead, the physician in charge contacts donors to obtain a second sample that is then sent to the Public Health State Laboratory for confirmation.

At CIB, all sera reactive by ELISA were confirmed by another technique, as per the Mexican Official Standard NOM-032-SSA-2010 and the Procedure Manual for Chagas Disease in Mexico. These guidelines recommend confirming a positive case with two serological techniques with different principles or antigenic preparations. Immunofluorescence was used in this study^20,21^.

### T. cruzi antigen

A total extract of *T*. *cruzi* Mexican isolate epimastigote from naturally infected *Triatoma dimidiata* were used as the antigen. *T. cruzi* isolates from the Yucatan peninsula (Camp-6, Camp-7, Camp-11, Camp-12, Camp-13, Calkini, Samula-1, CIET-1, Xcalotl-1, Xcalotl-2, Asilo-1, Asilo-2, and H-1); the Veracruz State (LJ02); the Nayarit State, western Mexico (N); and from Brazil (Y and CL-Brener) were used. The parasites were cultured in a liver-infused tryptose medium with 10% heat-inactivated fetal calf serum and were then collected during their exponential growth phase. Cells were washed thrice in phosphate buffered saline (pH 7.4) and pelleted. The pelleted cells were sonicated on a bed of ice and in a protease inhibitor (PMSF at 1 mM, EDTA at 5mM, and aprotinin at 1mM final concentration) for 10 s, eight times at 1 min intervals. The suspension was evaluated for total parasitic lysis and then centrifugated at 10,000 g for 30 min at 4 °C. The crude antigen isolate extracts were stored at −70 °C until further use.

### Electrophoresis and Western Blot

Overall, 50 micrograms per lane were loaded for each *T. cruzi* isolate. Gels (SDS-PAGE) at 10% and 4% acrylamide were used to obtain 10–250 kDa proteins. Electrophoresis was performed at 100 V for 2 h at room temperature and stained with Coomassie blue G250.

Western blot assays were carried out by transferring the proteins in the gels onto nitrocellulose membranes. The membrane of each *T. cruzi* isolate was left overnight in PBS containing 5% skimmed milk, under constant shaking, at 4 °C. The membrane was cut into strips and left overnight in PBS containing 5% skimmed milk, under constant shaking, at 4 °C. Each strip was incubated with the serum and diluted 1:500 in PBS containing 5% skimmed milk, for 2 h, at room temperature. After washing five times with 0.1% Tween 20 in PBS solution, the strips were incubated at room temperature with peroxidase-conjugated anti-human IgG at a 1:10,000 dilution for 2 h. After incubation, five washes were carried out and the reaction was developed by adding 0.5 mg/mL of 3,3-diaminobenzidine in citrate buffer and 0.02% hydrogen peroxide. Distilled water was used to stop the reaction. Positive and negative control sera and blanks were included in each assay.

### ELISA

**Figure 1:**
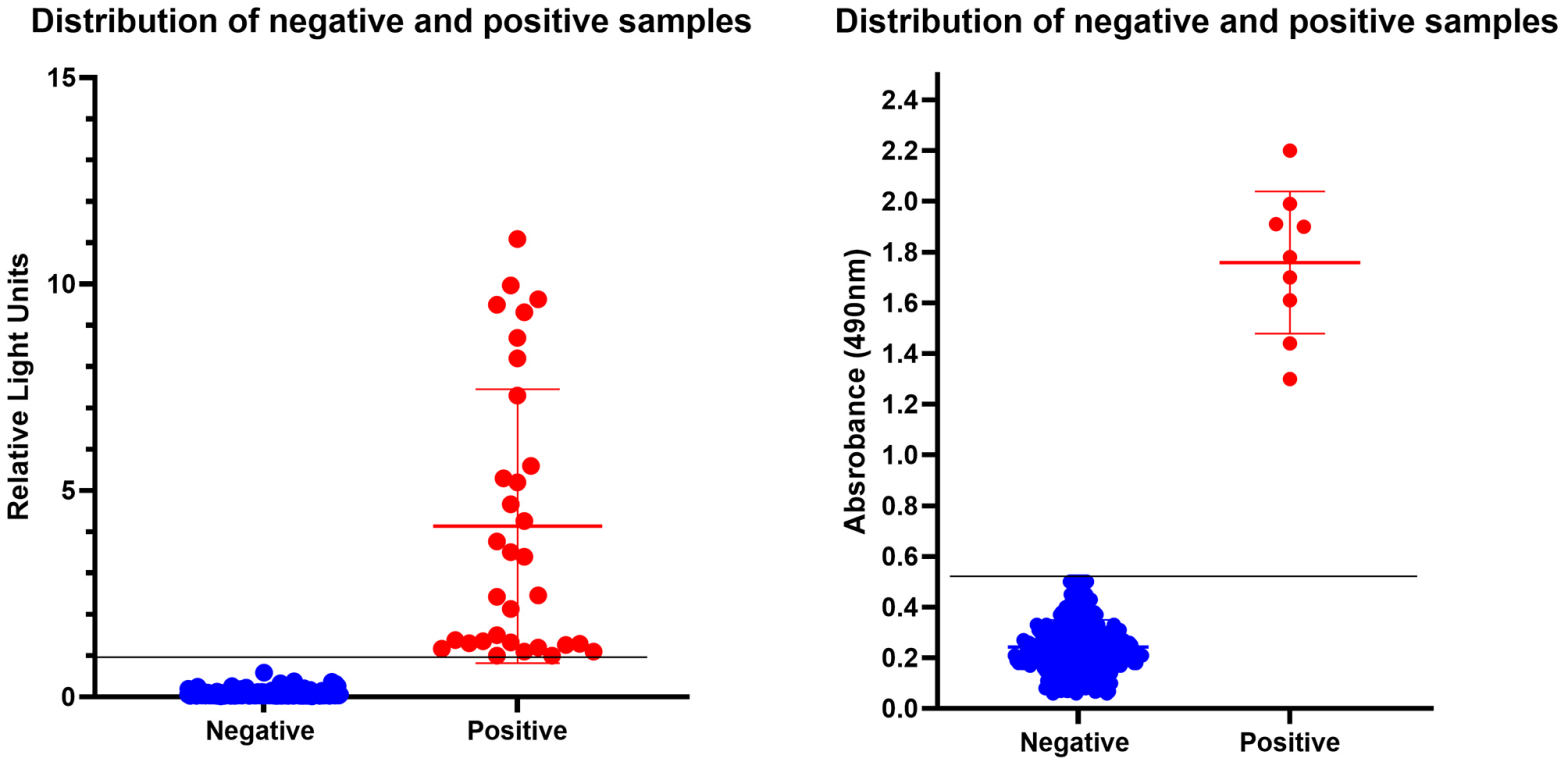
IgG anti-*Trypanosoma cruzi* in Mexican samples from the Yucatan peninsula and the cut-off values of two serological assays. In the left panel: the Architect System (Abbott) chemiluminescence assay with paramagnetic microparticles coupled to FP3, FP6, FP10, and TcF chimeric recombinant antigens to analyze 289 negative and 32 positive samples. Median relative light units (RLU) totaled 4.13 for positive blood donors and 0.07 (SD 0.06) for negative ones. The manufacturer’s cut-off for this assay is 1.0 RLU. In the right panel: an in-house ELISA with a local isolate of *T. cruzi* X-2 to analyze the distribution of 294 negative samples and nine samples from patients with CCC. The cut-off value (0.53) resulted from adding three standard deviations (SD) to the mean optical density (OD) of the seronegative healthy individuals.

The diagnosis of chronic Chagas disease was performed using two ELISAs. At CETS, the chimeric *Trypanosoma cruzi* antigens (ABBOTT PRISM Chagas assay) used paramagnetic microparticles coupled to FP3, FP6, FP10, and TcF chimeric recombinant antigens^22,23^. The manufacturer’s instructions were followed. Results are shown in relative light units (RLU). The cut-off is between RLU sample/RLU calibrant, in which samples >1 were positive results; <0.8 negative ones; and 0.8–1, inconclusive. The historical 2023-year sera from 289 healthy blood donors who were previously tested as negative for anti-*T. cruzi* antibodies 35 positive samples were graphed. Their median RLU in positive blood donors totaled 4.13 (Figure 1). A 98.4% sensitivity and a 98.7% specificity were established by the manufacturer^24^.

At CIB, an in-house ELISA with a crude antigen extract from regional *T. cruzi* X-2 isolate was used to screen donor samples. ELISA assays were carried out as reported^10^. In brief, a polystyrene plate (Nunc Polysorb Plates) was coated with 10 µg/mL of crude extract of *T*. *cruzi* in alkaline-buffered solution and incubated overnight at 4 °C. After blocking with PBS-Tween 20 0.05% and 3% skimmed milk for 1 h, samples were added at a dilution ratio of 1:500 and incubated for 2 h at room temperature. After washing, the reaction was incubated at room temperature with peroxidase-conjugated anti-human IgG diluted to1:10000 for 1 h. The reaction was developed by adding O-phenilen-diamine and reading at 490 nm in a plate reader. Sera from 289 healthy volunteers who had been tested as negative for anti-*T. cruzi* antibodies were analyzed for statistical distribution and to establish the assay cut-off value. The mean optical density (OD) of seronegative healthy individuals plus three standard deviations (SD) was set as the cut-off value. Sera samples of nine patients with CCC were used as positive controls. The cut-off was set 0.53. (Figure 1).

### Immunofluorescence

Immunofluorescence was carried out as reported. In brief, a drop of an epimastigote (X-2 regional isolate) suspension was air-dried on a glass slide. The sample was then diluted to 1:50 in PBS and incubated in a humidified chamber for 30 min. The slides were washed and incubated with conjugate anti-human IgG fluorescein for 30 min. After incubation, the slides were observed under an epifluorescence microscope. A green-colored fluorescence from the bodies of the parasites was considered a positive result. The degree of fluorescence for a positive result ranged from + to ++++, using a positive control as reference.

### Serum samples

The sera used were sourced from 1,500 blood donors from CETS, in Campeche, Mexico, from December 2023 to March 2024. Another 2,800 samples from young students from Universidad Autonoma de Campeche were collected from August 2022 to September 2022.

Serum samples from patients with CCC and from blood donors who had been validated as positive by two techniques were used to analyze the antigen profile of *T. cruzi* strains and isolates by Western blot assays and to select the best *T. cruzi* isolates for the in-house ELISA in this study.

### Statistical analysis

Descriptive frequencies and percentage were used to summarize the data. The Mann-Whitney and Student’s *t*-tests for numerical variable differences between groups were used. Results are shown as means ± standard errors, with p<0.05. A two-by-two contingency table was used to estimate test sensitivity and specificity, positive and negative predictive values, and kappa indices. Test Sensitivity = a/a+c (100); Specificity= d/b+d (100); Kappa index κ = 1−Pe/Po−Pe, in which Po = a+d/N; Pe = (a+b)(a+c)+(c+d)(b+d)/ N^2^. GraphPad Prism, version 10.2, was used for such calculations.

## RESULTS

### Electrophoresis and Western Blot

**Figure 2:**
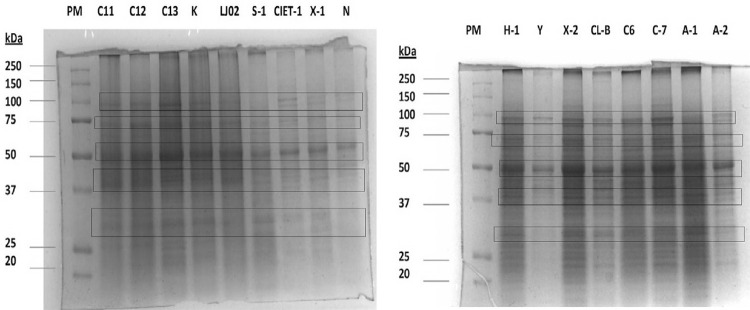
Protein profile of a *Trypanosoma cruzi* epimastigote extract: representative image of SDS-PAGE at 10% acrylamide of *T*. *cruzi* Mexican isolate epimastigote total extract. In total, thirteen *T. cruzi* isolates from the Yucatan peninsula (Camp-6, Camp-7, Camp-11, Camp-12, Camp-13, Calkini, Samula-1, CIET-1, Xcalotl-1, Xcalotl-2, Asilo-1, Asilo-2, and H-1), the Veracruz State (LJ02), Nayarit (N, western Mexico), and Brazil (Y and CL-Brener).

Analysis of the 10% SDS-PAGE gels for 17 *T. cruzi* extracts found a similar pattern in 13 *T. cruzi* crude extracts from the Yucatan peninsula (Campeche state) and from Nayarit (N), Veracruz (LJ-02), as well as the reference strains Y and CL-Brener. Clear and predominant bands emerged at 95-, 72-, 50-, 37-, and 30-KDa regions in all *T. cruzi* extracts. Even under the limited resolution of SDS-PAGE, this study observed bands above 50KDa in all eight gel bands, and below 50 KDa in up to 18 gel bands for all *T. cruzi* extracts (band intensity varied). However, results show a scarce number of bands above 100 KDa (Figure 2).

**Figure 3:**
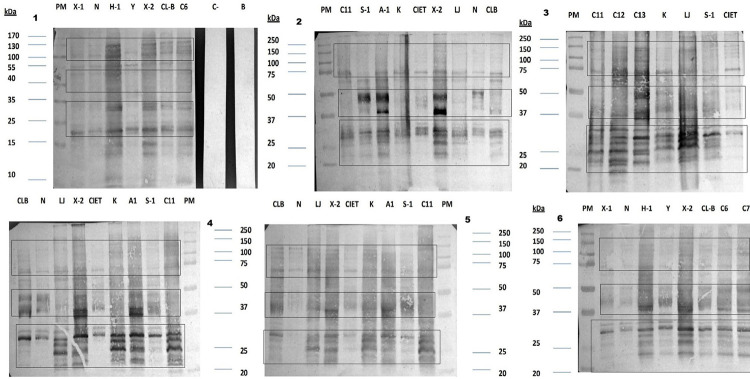
IgG reactivity of chronic chagasic myocardiopathy patients against a *Trypanosoma cruzi* epimastigote extract: representative image of a western blot of six sera samples (1 to 6) against *T*. *cruzi* Mexican isolate epimastigote total extract. *T. cruzi* isolates from the Yucatan peninsula (X-1, H-1, X-2, C6, C11, S-1, A-1, K, CIET, C12, C13), the Veracruz State (LJ02), Nayarit (N, western Mexico), and Brazil (Y and CL-Brener). Upper (72-150KDa), middle (37-50KDa), and bottom (18-35KDa).

To analyze immunodominant antigens, this study tested the immunoblot of previous *T. cruzi* extracts with six serum samples of confirmed CCC cases. Firstly, each serum sample obtained similar patterns of antigenic proteins to several *T. cruzi* antigenic extracts, despite the antigenic profile of each serum sample, they showed common protein antigens with varying antigenic differences in *T. cruzi* isolates. We separated antigenic profiles into three regions: upper (72–150KDa), middle (37–50KDa), and bottom (18–35KDa). In the upper region, the antigenic pattern showed greater homogeneity between serum samples to the *T. cruzi* isolates, except for serum 3, which shared several immunodominant proteins. In the middle region, the pattern showed greater heterogeneity between serum samples and some stronger immunodominant antigens. The bottom region generally included more immunodominant antigens in all serum samples (Figure 3). Data indicate that any *T. cruzi* extracts may serve the diagnosis of Yucatan CCC patients, even with *T. cruzi* extracts from other Mexican regions or Brazil. Although some *T. cruzi* isolates reacted less intensely than others (such as the Nayarit, CIET, and X-1 isolates), all *T. cruzi* isolates showed common immunodominant antigens.

To determine immunodominant antigens via indeterminate Chagas disease subjects, we used three serum samples from *T. cruzi*-positive blood donors that had been validated by two techniques. We chose representative serum samples with high, medium, and low intensity reactions in ELISA assays for this investigation.

**Figure 4:**
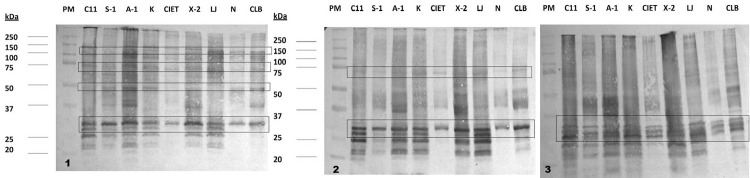
IgG reactivity of blood donors against an extract of *Trypanosoma cruzi* epimastigotes: representative image of a western blot of three sera samples (1 to 3) of blood donors positive against *T*. *cruzi* Mexican isolate epimastigote total extract. *T. cruzi* isolates from the Yucatan peninsula (C11, S-1, A-1, K, CIET, X-2), the Veracruz State (LJ02), Nayarit (N, western Mexico), and Brazil (CL-Brener). Upper (72-150KDa), middle (37-50KDa), and bottom (18-35KDa).

Immunoblot analysis showed that blood donors showed fewer antigenic bands than CCC patients and that the main immunodominant bands occurred in the low molecular weight region (20–32 KDa). However, these antigens occurred in all *T. cruzi* isolates in this study (Figure 4). These data guided our selection of the most suitable *T. cruzi* regional isolates for blood bank screening via in-house ELISA assays.

### Blood bank donors seroprevalence

CETS constitutes the main blood bank for Campeche State, Mexico. Its blood donors live in urban and rural communities. In the historical data from 2023 and 2024, CETS had 19,296 donors, of which 35 tested positives for Chagas disease by chimeric *Trypanosoma cruzi* antigens **(**ABBOTT PRISM Chagas assay), a 0.18% seroprevalence. Figure 1 shows the graphic distribution of reaction intensity in RLU, evincing a mean of 4.13 RLU for the positive samples.

CETS found three positive samples out of 1,500 (0.2%) via chimeric *Trypanosoma cruzi* antigens (ABBOTT PRISM Chagas assay), whereas the in-house ELISA found four out of 1,500 (0.26%). Only one donor sample tested positive in both laboratories, whereas 1,494 samples tested negative at both labs. As no laboratory has a gold standard, we considered CETS as the best proxy reference standard, followed by CIB. Using a two-by-two contingency table and CETS as reference, CIB sensitivity totaled 33% (95%CI 0.01710 to 0.8815); specificity, 99.8% (95%CI 0.9941 to 0.9995); positive predictive value, 25% (95%CI 0.01282 to 0.6994); high negative predictive value, 99.8% (95%CI 0.9951 to 0.9998); and the kappa index, 0.2.

Immunofluorescence found one positive result out of three by chimeric *Trypanosoma cruzi* antigens (ABBOTT PRISM Chagas assay), and the in-house CIB ELISA found three out of four positives with the regional *T. cruzi* crude extract. The overall seropositive sample totaled three out of 1,500 for (0.2% seroprevalence). The higher agreement between immunofluorescence and the in-house ELISA may stem from both assays using the same *T. cruzi* isolate and having serum samples from the same regional area.

As *Triatoma dimidiata* configures the main vector in Yucatan peninsula and due to the local population’s familiarity with insect vector, we screened 2,800 young healthy subjects from Universidad Autonoma Campeche for exposure to *T. cruzi* infection. ELISA results showed four positive samples out of 2,500 (0.14% seroprevalence), and immunofluorescence confirmed two samples (0.07% seroprevalence) (Table 1).

**Table 1:** Demographic data and seroprevalence among blood donors

Case	Age/gender	Place of residence	Chimeric *Trypanosoma cruzi* antigens (ABBOTT PRISM Chagas assay) (RLU)	ELISA local antigen (OD)	Immunofluorescence local antigens	Result
1	32/male	Urban	0.3	0.67	Positive 1:50	Positive
2	34/female	Urban	13	0.44	Negative	Negative
3	50/male	Urban	9.29	0.83	Positive 1:50	Positive
4	57/male	Urban	0.4	0.62	Positive 1:50	Positive
5	27/male	Rural	0.4	0.60	Negative	Negative
6	21/female	Rural	9.7	0.32	Negative	Negative

Abbott PRISM Chagas chemiluminescent assay results are shown as relative light units (RLU); cut-off >1 positive; ELISA local antigen results are shown as optical density at 492nm cut-off >0.53 positive; Immunofluorescence cut-off dilution is 1:50.

## DISCUSSION

*T. cruzi* isolate and antigen source variability and human population heterogeneity could explain the variability in intensities observed in the reactions to antigens during serodiagnosis. Many studies have analyzed this issue^3-5,8-11^ and some have suggested inclusive variation in the sensitivity and specificity of recombinant antigens used for diagnosis. A recent report has described the poor performance of diagnostic tests in samples from Central American and Mexican donors^3^. Our SDS-PAGE analysis of Yucatan *T. cruzi* extracts shows common proteins in the *T. cruzi* isolates, including with those in the reference CL-Brener and Y strains. Specifically, proteins bands at 30, 40, 72, and 95 KDa occurred the most often and consistently in all extracts. Western blot analysis showed strong and immunodominant antigens at 20, 25, 32, 37, 72, 75, 100, and 150 KDa (identified with serum samples of Yucatan CCC patients). Previous studies have reported similar antigenic profiles with another Mexican *T. cruzi* isolate and CCC serum samples^25,26^. Note that the serum samples of blood donors in this study preferentially showed antigens below 32 KDa, whereas CCC ones showed antigens with a broad range of molecular weights. These data support the hypothetical possibility of performing diagnoses with several *T. cruzi* isolates as they share common proteins and antigens. Regarding our in-home ELISA, the X-2 isolate may represent local immunodominant antigens and reference Brazilian strains.

The Chagas seroprevalence in blood donors equaled 0.2% in the chimeric *Trypanosoma cruzi* antigens (ABBOTT PRISM Chagas assay) and 0.26% in the in-house ELISA assay with local *T. cruzi* isolate antigenic extracts. Considering two positive concordant tests, seroprevalence total 0.2%, resembling those in blood banks from larger Mexican metropolitan municipalities, in which *T. dimidiata* vector remains uncommon^17,18^. Our data suggest a low similar risk of blood donors infected with *T. cruzi* in blood banks in larger Mexican urban areas and those in which *T. dimidiata* is common.

The low sensitivity in our study (33%) contrasts with its high specificity (99.8%). A possible explanation lies in the characteristics of the chimeric *Trypanosoma cruzi* antigens in the ABBOTT PRISM Chagas assay. Its Food and Drug Administration license details a study involving 16,249 serum specimens from volunteer blood donors in non-endemic regions at three blood centers in the United States. The initial screening found 26/16,249 (0.16%) reactive samples, but RIPA testing only confirmed three of these as true positives, representing a mere 12% confirmation rate^24^. This evidence suggests that the chimeric *Trypanosoma cruzi* antigens (ABBOTT PRISM Chagas assay) may generate a significant number of false positives if used as a primary screening tool.

Recent publications reported a 90.7% sensitivity with the chimeric *Trypanosoma cruzi* antigens **(**ABBOTT PRISM Chagas assay) in a panel of 86 positive and 262 negatives Mexican sera. They also found that chimeric *Trypanosoma cruzi* antigens (ABBOTT PRISM Chagas assay) showed a median RLU of 8.92 in samples from South American donors, 7.97 in Central American donors, and 5.09 in Mexican donors^3^. Our analysis obtained a slightly lower median RLU (4.13). This finding may be explained by the low antibody titers in our population. Subjects living in high endemic regions show higher antibody titers than those in low endemic regions^27,28^. The source of *T. cruzi* strains may also affect reaction intensity (i.e., sensitivity), failing to change its positive (i.e., specificity) status^10^. A further potential reason for this discrepancy could refer to the insufficient number of positive samples in our analysis (three), which inherently lowered the kappa index and sensitivity. More precise estimations of concordance between tests require a larger cohort with an increased and more balanced representation of positive and negative specimens.

Moreover, the screening techniques in blood banks show a high number of reactive sera, a number that substantially decreases upon confirmation with a second technique. One study evaluated 107,321 blood donors via ELISA with an EVOLIS™ automated microplate processor (BIO-RAD, CA, US) and 402,726 donors via the Chimeric *Trypanosoma cruzi* antigens (ABBOTT PRISM Chagas assay) screening test. The authors reported 2,613 reactive donors in screening tests, but the Micro-ELISA confirmatory test (the Chagascreen Plus kit) (BIO-RAD, CA, US) showed 595 true positives (22.7%)^18^. Another study used ELISA for screening and immunofluorescence as the confirmatory assay, they found 277 reactive blood samples out of 53,941 of which 129 was positive in confirmatory test (46%)^29^. A recently published paper reported 261 reactive samples according to an ELISA screening assay, confirming 92 by immunofluorescence (35%)^30^. A blood bank in Brazil reported 1,982 reactive blood donors out of 608,353 via screening chemiluminescence, confirming 602 positive results (30.3%)^31^. These data suggest that screening assays show high sensitivity but their low specificity unnecessarily discards numerous blood units, directly impacting blood supply in blood banks.

Blood banks in Mexico City, a large urban metropolis, reported a 0.12% seroprevalence for *T. cruzi* antibodies. In contrast, an area endemic for the *T. dimidiata* vector showed a slightly higher seroprevalence (0.2%). This suggests that while *T. dimidiata* may not be a highly efficient vector for *T. cruzi* transmission, the migration of infected individuals from endemic regions to large urban settings likely contributes to the seroprevalence in this large urban metropolis.

The seroprevalence of antibodies against *T. cruzi* in young healthy students showed four positive samples out of 2,500 (a 0.14% seroprevalence) via ELISA, confirming two samples by immunofluorescence (a 0.07% seroprevalence).

This low seroprevalence agrees with studies in young people and in rural inhabitants for the same area^16,26^. Although the Yucatan peninsula configures an endemic area for *T. cruzi* infection where *T. dimidiata* constitutes the main vector, seroprevalence in humans remains low^12,13^.

## CONCLUSION

Seroprevalence of *T. cruzi* antibodies in blood donors and young people in Campeche State, Mexico, can be considered low despite the ubiquity of *T. dimidiata*. The use of commercial serodiagnosis platform with recombinant antigens from non-Mexican *T. cruzi* strains is comparable to local crude *T. cruzi* extracts in its specificity. Nevertheless, improved sensitivity requires further efforts. Anti-*T. cruzi* antibody seroprevalence in a blood bank within a *Triatoma dimidiata* endemic region was comparable to seroprevalence in blood banks in large municipalities.

## References

[1] Shikanai-Yasuda MA (2022). Emerging and reemerging forms of Trypanosoma cruzi transmission. Mem Inst Oswaldo Cruz.

[2] Pan American Health Organization Guidelines for the diagnosis and treatment of Chagas disease.

[3] Kelly EA, Bulman CA, Gunderson EL, Irish AM, Townsend RL, Sakanari JA (2021). Comparative performance of latest-generation and FDA-cleared serology tests for the diagnosis of Chagas disease. J Clin Microbiol.

[4] Whitman JD, Bulman CA, Gunderson EL, Irish AM, Townsend RL, Stramer SL (2019). Chagas disease serological test performance in U.S. blood donor specimens. J Clin Microbiol.

[5] Verani JR, Seitz A, Gilman RH, LaFuente C, Galdos-Cardenas G, Kawai V (2009). Geographic variation in the sensitivity of recombinant antigen-based rapid tests for chronic Trypanosoma cruzi infection. Am J Trop Med Hyg.

[6] Wiener Laboratorios Chagatest ELISA recombinante v.3.0.

[7] Wiener Laboratorios Chagatest ELISA recombinante v.4.0.

[8] Risso MG, Sartor PA, Burgos JM, Briceño L, Rodríguez EM, Guhl F (2011). Immunological identification of Trypanosoma cruzi lineages in human infection along the endemic area. Am J Trop Med Hyg.

[9] Ballinas-Verdugo M, Reyes PA, Mejia-Dominguez A, López R, Matta V, Monteón V (2011). Enzyme-linked immunosorbent assay and polymerase chain reaction performance using Mexican and Guatemalan discrete typing unit I strains of Trypanosoma cruzi. Vector Borne Zoonotic Dis.

[10] Luquetti AO, Espinoza B, Martínez I, Hernández-Becerril N, Ponce C, Ponce E (2009). Performance levels of four Latin American laboratories for the serodiagnosis of Chagas disease in Mexican sera samples. Mem Inst Oswaldo Cruz.

[11] Monteón V, Triana-Chávez O, Mejía-Jaramillo A, Pennignton P, Ramos-Ligonio A, Acosta K (2014). Circulation of Tc Ia discrete type unit Trypanosoma cruzi in Yucatan Mexico. J Parasit Dis.

[12] Moo-Millan JI, Hernández-Andrade A, May-Concha IJ, Montalvo-Balam TJ, Arnal A, Talavera-Escalante J (2023). Temporal variation of Triatoma dimidiata abundance and infection with Trypanosoma cruzi in domestic and sylvatic habitats of rural Yucatan, Mexico. Acta Trop.

[13] Reyes-Novelo, Ruiz-Piña H, Escobedo-Ortegón J, Barrera-Pérez M, Manrique-Saide P, Rodríguez-Vivas R (2013). Triatoma dimidiata (Latreille) abundance and infection with Trypanosoma cruzi in a rural community of Yucatan, Mexico. Neotrop Entomol.

[14] Monteón V, Solis-Oviedo R, Lopez R, Hernández O, Tellez CA (2015). Low seroprevalence of Trypanosoma cruzi infection and chronic chagasic cardiomyopathy in a region with abundance of triatomine vectors in Yucatan Peninsula of Mexico. Ann Parasitol.

[15] Alducin-Téllez C, Rueda-Villegas E, Medina-Yerbes I, Hernández O, López R, Peña-Hernández V (2011). Prevalencia de serología positiva para Trypanosoma cruzi en pacientes con diagnóstico clínico de miocardiopatía dilatada en el Estado de Campeche, México. Arch Cardiol Mex.

[16] Monteon V, Alducin C, Hernández J, Ramos-Ligonio A, Lopez R (2013). High frequency of human blood in Triatoma dimidiata captured inside dwellings in a rural community in the Yucatan Peninsula, Mexico, but low antibody seroprevalence and electrocardiographic findings compatible with Chagas disease in humans. Am J Trop Med Hyg.

[17] González-Guzmán S, González-Cano P, Bagu ET, Vázquez-Vega S, Martínez-Salazar M, Juárez-Montiel M (2022). Seroprevalence of Trypanosoma cruzi in eight blood banks in Mexico. Arch Med Res.

[18] González-Guzmán S, Paredes-Cervantes V, Bagu ET, Crescencio-Trujillo JA, Guerra-Marquez A, Rivas N (2019). Seroprevalence and geographical distribution of sero-positive blood donors to Trypanosoma cruzi at the central blood bank of the National Medical Center "La Raza". Transfusion.

[19] García-Montalvo B (2011). Trypanosoma cruzi antibodies in blood donors in Yucatan state, Mexico. Rev Med Inst Mex Seguro Soc.

[20] México, Secretaría de Salud Manual de diagnóstico y tratamiento de la enfermedad de Chagas.

[21] México, Secretaría de Salud, Subsecretaría de Prevención y Promoción a la Salud, Centro Nacional de Programas Preventivos y Control de Enfermedades (2019). Programas de Enfermedades Transmitidas por Vectores. Manual de procedimientos para la enfermedad de Chagas en México.

[22] Chang CD, Cheng KY, Jiang LX, Salbilla VA, Haller AS, Yem AW (2006). Evaluation of a prototype Trypanosoma cruzi antibody assay with recombinant antigens on a fully automated chemiluminescence analyzer for blood donor screening. Transfusion.

[23] Cheng KY, Chang CD, Salbilla VA, Kirchhoff LV, Leiby DA, Schochetman G (2007). Immunoblot assay using recombinant antigens as a supplemental test to confirm the presence of antibodies to Trypanosoma cruzi. Clin Vaccine Immunol.

[24] ABBOTT PRISM Trypanosoma cruzi (E coli, recombinant) antigen.

[25] Torres-Gutiérrez E, Barrios-Palacios D, Ruiz-Hernández A, Cabrera-Bravo M, Guevara-Gómez Y, Rojas-Wastavino G (2015). Identificación de componentes inmunodominantes de un aislado de Trypanosoma cruzi por inmunoblot y su estandarización con fines diagnósticos. Gac Med Mex.

[26] Cervantes-Landín A, Martínez I, Schabib M, Espinoza B (2014). High molecular weight proteins of Trypanosoma cruzi reduce cross-reaction with Leishmania spp. in serological diagnosis tests. Biomed Res Int.

[27] Balan LU, Yerbes IM, Piña MA, Balmes J, Pascual A, Hernández O (2011). Higher seroprevalence of Trypanosoma cruzi infection in dogs than in humans in an urban area of Campeche, Mexico. Vector Borne Zoonotic Dis.

[28] Sosa-Jurado F, Mazariego-Aranda M, Hernández-Becerril N, Garza-Murillo V, Cárdenas M, Reyes PA (2003). Electrocardiographic findings in Mexican chagasic subjects living in high and low endemic regions of Trypanosoma cruzi infection. Mem Inst Oswaldo Cruz.

[29] Lopes PS, Ramos EL, Gómez-Hernández C, Ferreira GL, Rezende-Oliveira K (2015). Prevalence of Chagas disease among blood donor candidates in Triangulo Mineiro, Minas Gerais State, Brazil. Rev Inst Med Trop Sao Paulo.

[30] Beltrán-Durán M, Hilarión-Gaitán LB, Berrío-Pérez M, Bermúdez MI (2017). Detección de anticuerpos para Trypanosoma cruzi en donantes de sangre. Caquetá, Colombia, 1995 a 2010. Rev Salud Publica (Bogota).

[31] Costa AC, Rocha EA, Silva JD, Fidalgo AS, Nunes FM, Viana CE (2020). Prevalence of Trypanosoma Cruzi infection in blood donors. Arq Bras Cardiol.

